# Experience of child welfare services and long-term adult mental health outcomes: a scoping review

**DOI:** 10.1007/s00127-021-02069-x

**Published:** 2021-03-29

**Authors:** Sarah McKenna, Michael Donnelly, Ifeoma N. Onyeka, Dermot O’Reilly, Aideen Maguire

**Affiliations:** 1 Centre for Public Health, Queen’s University Belfast, Institute of Clinical Sciences B, Royal Hospitals Site, Belfast, BT12 6BJ Northern Ireland UK; 2 Administrative Data Research Centre Northern Ireland (ADRC-NI), Belfast, UK

**Keywords:** Child welfare services, Adult mental health status, Scoping review

## Abstract

**Purpose:**

This is the first comprehensive review of empirical research that investigated the association between receipt of child welfare services and adult mental health outcomes. The review summarised the results of studies about mental health outcomes of adults with a history of child welfare involvement.

**Methods:**

A scoping review methodology was used to search five electronic databases (MEDLINE, EMBASE, PsychINFO, IBSS, Social Policy and Practice). Studies were included if they examined any child welfare exposure (including receipt of services while remaining at home/being placed in care) and adult mental health status.

**Results:**

In total 4591 records were retrieved, of which 55 met the eligibility criteria. Overall, receipt of child welfare services was associated with an increased risk of adult mental ill-health, suicide attempt and completed suicide. Results regarding potential moderating factors, such as gender and care-related experiences, were mixed. Relatively few studies investigated the reasons for requiring child welfare services, the experience of abuse or neglect or the adult outcomes of child welfare service users who remained in their own homes. Mental ill-health was defined and measured heterogeneously and details about the nature and type of welfare service utilisation were lacking.

**Conclusion:**

There is a need for detailed, longitudinal studies to better understand the relative contribution of pre-existing adversity versus experiences during and after exposure to child welfare services on adult mental health outcomes. More standardised measures of mental ill-health and greater detail from authors on specific care exposure are also needed.

**Supplementary Information:**

The online version contains supplementary material available at 10.1007/s00127-021-02069-x.

## Introduction

It is the statutory responsibility of child welfare systems to protect the safety and wellbeing of children in need of help or protection. These social care systems have differing names and orientations across countries and responsibilities include both family support and child protection measures [1]. Where there are serious concerns about safety, a child may be removed from their birth family and placed in residential care, kinship care (out-of-home care with members of the biological family) or foster care (out-of-home care with strangers) [2, 3]. Previous research has focused predominately on children in out-of-home care (OHC), suggesting they are at an increased risk of a range of mental health disorders and suicide attempt compared with their general population peers whilst in care [4–8]. Evidence of poor outcomes across numerous domains for care experienced children has fuelled debate around the efficacy of OHC versus in-home care (IHC) [9–13]. As the number of children in the child welfare system has increased in many countries, it is of key policy and practice importance to examine the nature and extent to which these mental health risks persist into adulthood and to explore variation in outcomes based on the type of child welfare service received [14–19].

The proliferation of research in this area is challenging to navigate and few studies have attempted to provide an overview. Two systematic reviews have explored long-term health and social outcomes associated with OHC [20, 21]. Both reported elevated risk of mental-ill health for adults with a care history compared to the general population, though they touched only briefly on mental health and did not examine in detail how adult mental health outcomes may vary as a function of placement type or experiences during care. The first reviewed studies published 2004–2015 categorised into two groups according to the child welfare orientation of the country (child protection vs family service) [20]. Adults with a history of OHC from both welfare systems had higher rates of mental health disorder than their general population peers, with women faring worse than men. It did not, however, report any findings related to suicide attempt or suicide mortality [20]. The second reviewed all studies published 2000–2016 limited to Nordic countries [21]. Measured in terms of psychiatric diagnoses, uptake of psychotropic medication and hospital admissions, the review concluded that adults with a history of OHC in Nordic countries were at greater risk of mild and severe mental health problems compared to the general population [21]. No systematic review exists which exclusively explores the long-term adult mental health outcomes of individuals in receipt of child welfare services as children. The current study aims to do that and extends the scope of previous reviews by: (i) including adults who received in-home care, (ii) synthesising evidence on placement type and care experiences associated with adult mental health, (iii) setting no geographical limitations and (iv) including studies from an extended timeframe (1995–2019).

Initial exploratory searches indicated that a diverse body of literature on child welfare services and adult mental health exists. As systematic reviews are characterised by a more narrowly defined focus and of interest in this review was all types of exposure to child welfare services and mental health, a scoping review methodology was chosen. A scoping review provides a comprehensive, overarching and rigorous approach to marshalling the available literature where mapping the breadth of evidence on a subject is the primary aim [22]. The research questions were:

1. What is the scope, nature and diversity of the empirical literature on the topic and where are the gaps?
2. What is the relationship between receipt of child welfare services and adult mental health status?

## Methods

This study used the scoping review methodology outlined by the Joanna Briggs Institute which incorporates best practice developed by Arksey and O’Malley and Levac et al. [23–25]. Key stages of a scoping review are: (1) identifying the research question; (2) identifying relevant studies; (3) study selection; (4) charting the data; (5) collating, summarising and reporting the results and (6) consultation (optional). Reporting follows the preferred reporting items for systematic reviews and meta-analyses extension for scoping reviews (PRISMA-ScR) guidelines [26].

### Search strategy

MEDLINE, EMBASE, PsychINFO, the International Bibliography of the Social Sciences (IBSS) and Social Policy and Practice were searched for studies between 1 January 1995 and 29 April 2019 using keywords and index headings adapted to the search functions of each database (supplementary Table TS1.) Date parameters were chosen to correspond with widespread changes globally in child welfare policies and practices since the mid-1990s and studies published prior to 1995 have been explored elsewhere [27]. Reference lists of eligible studies were screened for additional publications. Two reviewers independently screened the titles and abstracts of all the publications and any discrepancies were resolved by a third reviewer. Full-text assessment was carried out by the lead author and a random 20% sample of final papers were checked by a second reviewer.

### Inclusion and exclusion criteria

Eligibility criteria were specified using the population, concept, context approach recommended for scoping reviews [22, 23].

*Population:* Individuals aged 16 years + with experience of IHC or OHC at any point during childhood or adolescence. Individuals still receiving care, adoptees, institutional abuse survivors and indigenous populations placed in OHC on race grounds were excluded.

*Concept:* Studies that examined the adult mental health of individuals exposed to child welfare services (follow-up at age 16 years or older as it is possible to leave care at age 16). Services included OHC (foster care by non-family, kinship foster care or residential care) or IHC (e.g. child protection or family support services). Services that were not ‘care as usual’, such as Multi-treatment Foster Care or other treatment interventions, were excluded. Mental health outcomes included mental disorders, psychological conditions, mental health service use, suicide ideation or attempt and suicide mortality. Lifetime measures of mental health were excluded due to issues of temporality. Substance abuse or eating disorders were excluded due to the rarity and complexity of these mental disorders, i.e. eating disorders are difficult to diagnose and substance abuse as distinct from substance use is difficult to ascertain and can be both a symptom of and a cause of mental ill-health [28, 29].

*Context*. All geographic locations were included as were Government or independent research reports. English language publications only were eligible due to resource constraints.

### Data charting

The template data extraction table for scoping reviews recommended by the Joanna Briggs Institute was adapted [23]. Data were extracted based on the following fields: citation details; country; publication type; study design and data source; service type and comparator (if any); sample characteristics; mental health outcome; measurement method; covariates and key results. Numerical analysis, i.e. simple frequency counting within data fields was used to help summarise general findings. Narrative synthesis was used to report the main results. Type of exposure to child welfare services was taken as the primary conceptual category. Studies have examined what is known about OHC and IHC and adult mental health outcomes (i) individually, (ii) in comparison to each other and (iii) by OHC placement type. Where included studies examined additional exposures (e.g. adoption) or outcomes (e.g. substance abuse disorders, convictions or all-cause mortality) that were outside the scope of the review, these data were not extracted.

## Results

**Fig. 1:**
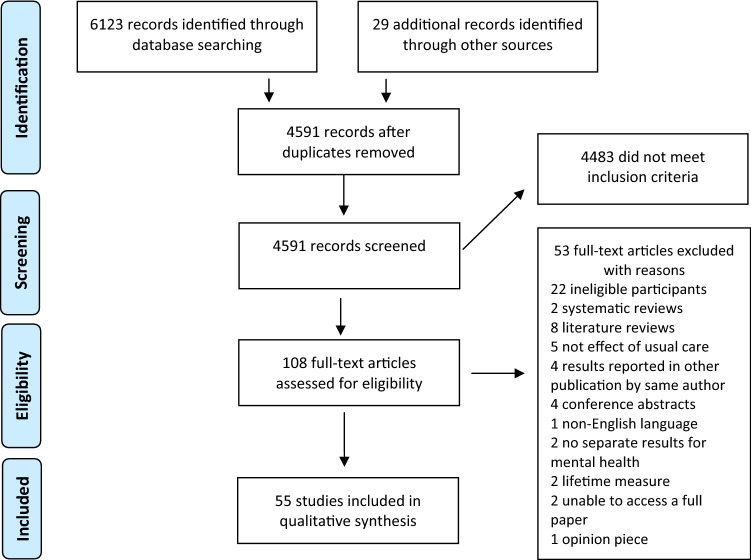
Flowchart of the study selection process

The search identified 4591 citations after the removal of duplicates. Following title and abstract screening, 108 titles remained for full-text assessment. At this stage, the two systematic reviews of OHC and adult outcomes mentioned above and eight literature reviews were excluded as any eligible individual studies they reported were already captured in the search strategy [20, 21, 30–37]. This review identified an additional 40 studies of OHC and adult mental health not included in earlier systematic reviews and to our knowledge is the first to include the literature on IHC. The search and selection process is presented in Fig. 1.

Fifty-five publications were included in this scoping review (Tables 1, 2, 3, 4). Studies were heterogeneous in terms of country, type of welfare service studied, sample characteristics, definition of mental health outcome, length of follow-up, measurement method and covariates included. Twenty-five studies were conducted in the USA, eleven in the UK, thirteen in Sweden, four in Canada, one in Finland and one in the Netherlands. Forty-eight were peer reviewed articles and seven were reports. Twenty-five used cross-sectional designs (two used baseline data from randomised control trials) and 30 were prospective longitudinal studies. Cohort sizes ranged from 37 to 1,002,122.

**Table 1:** Included studies of OHC and adult mental health

Study	Design *Data source*	Sample	Sample description	*N*	Mental health outcome	Measure	Covariates†	Summary of main results
Anctil et al. (2007), USA	Cross-sectional *Casey National Alumni Study*	Child welfare population	Adults in Casey OHC ≥ 1 year with documented mental or physical impairment as a child *F* = 50.9%	564	Overall mental health No. of diagnoses**	SF-12 CIDI	ACEs Demographics Care experiences	OHC predictors of overall mental health: *Relational services model* Age first placement *b* = − 4.1 (*p* = *ns*) Intensity of placement change *b* = 0.78 (*p* = *ns*) Felt loved *b* = 1.78 (*p* = *ns*) Helpfulness of foster parents *b* = − 0.99 (*p* = *ns*) Close adult relationship *b* = 0.51 (*p* = *ns*) Mental health services *b* = − 0.50 (*p* = *ns*) *Educational/occupational services model* Age at first placement *b* = − 0.51 (*p* = *ns*) Intensity of placement change *b* = 0.96 (*p* = *ns*) Employment training *b* = 0.43 (*p* = *ns*) Tutoring services *b* = − 0.85 (*p* = *ns*) Independent living services *b* = − 1.50 (*p* < 0.01) Special education *b* = − 0.21 (*p* = *ns*) Gifted education *b* = 0.51 (*p* = *ns*) OHC predictors of higher no. diagnoses: *Relational services model* Age at first placement *b* = − 0.03 (*p* = *ns*) Intensity of placement change *b* = 0.19 (*p* < .05) Felt loved *b* = 0.27 (*p* = *ns*) Helpfulness of foster parents *b* = 0.07 (*p* = *ns*) Close adult relationship *b* = 0.12 (*p* = *ns*) Mental health services: *b* = 0.14 (*p* < 0.01) *Educational/occupational services model* Age at first placement *b* = − 0.04 (*p* = *ns*) Intensity of placement change: *b* = 0.21 (*p* < .05) Employment training *b* = − 0.03 (*p* = *ns*) Tutoring services *b* = − 0.02 (*p* = *ns*) Independent living services *b* = − 0.04 (*p* = *ns*) Special education *b* = 0.03 (*p* = *ns*) Gifted education *b* = − 0.28 (*p* = *ns*)
Björkenstam et al. (2017), Sweden	Prospective cohort *Linked national registers*	Birth cohorts (1984–1988)	Young adults who experienced childhood adversities (CAs) including OHC/respite care < age 12 years and young adults with no CAs *F* = 48.7%	478,141	Depression	Psychiatric in/outpatient Prescribed antidepressants	ACEs Demographics Socio-economic	Risk clinical diagnosis OHC vs no childhood adversity: HR = 1.51 (95% CI 1.39,1.63) Risk antidepressant medication OHC vs no childhood adversity: HR = 1.07 (95% CI 1.00, 1.14)
Brännström et al. (2017), Sweden	Prospective cohort *Stockholm Birth Cohort*	Birth cohort (1953)	Adults placed in OHC due to family circumstances and adults with no history of OHC *F* = 50.3%	12,737	Mental health problems**	Psychiatric inpatient	Demographics Socio-economic	Likelihood of most disadvantaged outcome profile (incl. more mental health problems) OHC vs no OHC: Both genders: OR = 2.47 (95% CI 1.93, 3.15) M: OR = 2.06 (95% CI 1.45, 2.93) F: OR = 2.97 (95% CI 2.11, 4.19)
Bruskas and Tessin (2013), USA	Cross-sectional *Survey*	Child welfare population	Women with a childhood history of OHC *F* = 100.0%	101	Psychological distress Depression PTSD Service use	GHQ-12 Self-report	ACEs Care experiences	OHC predictors of psychological distress: ACEs during OHC: *b* = 0.35 (*p* = 0.15) No. of placements: *b* = − 0.10 (*p* = 0.18)
Buchanan (1999), UK	Prospective cohort *National Child Development Study*	Birth cohort (1958)	Adults with and without a history of OHC *F* = 50.0%	Unclear	Psychological problems Depression	Rutter 'A' Malaise Inventory		Psychological problems age 16: up to 25% of OHC group, higher than other family types Depression age 33: up to one in five of OHC group, exceeded by rate among women brought up in disadvantaged homes
Buchanan et al. (2000), UK	Prospective cohort *National Child Development Study*	Birth cohort (1958)	Adults with and without a history of OHC, including: 1. Care/birth (any OHC, living with birth family at age 16) 2. Care/restructured (any OHC, living in a restructured family at age 16) 3. Birth (brought up by birth parents, no OHC) *F* = nr	8441	Psychological problems Depression tendency	Rutter 'A' Malaise Inventory	Socio-economic Other	Likelihood psychological problem age 16 OHC vs no OHC: Care/birth F: OR = 1.8 (*p* = *ns*), M: OR = 5.0 (*p* < .001) Care/restructured F: OR = 5.1 (*p* < .001), M: OR = 3.0 (*p* < .05) Likelihood depression tendency age 33 OHC vs no OHC: Care/birth F: OR = 1.6 (*p* = *ns*), M: OR = 3.9 (*p* < .01) Care/restructured F: OR = 0.3 (*p* = *ns*), M: OR = 3.8 (*p* < 0.05)
Cheung and Buchanan (1997), UK	Prospective cohort *National Child Development Study*	Birth cohort (1958)	Adults with and without a history of OHC *F* = nr	12,537	Depression tendency	Malaise Inventory	Demographics Socio-economic	Likelihood depression tendency age 23 OHC vs no OHC: Both genders: OR = 2.69 (95% CI 1.89, 3.83) M: OR = 3.33 (95% CI 1.84, 5.98) F: OR = 2.45 (95% CI 1.60, 3.79) Likelihood depression tendency age 33 OHC vs no OHC: Both genders: OR = 2.29 (95% CI 1.46, 3.60) M: OR = 5.08 (95% CI 2.70, 9.46) F: OR = 1.27 (95% CI 0.65, 2.48)
Cooley et al. (2018), USA	Cross-sectional *Survey*	Child welfare population and a comparison sample	Adults with and without a history of OHC recruited from an urban primary care clinic for low income populations *F* = 35.2%	125	Depression Anxiety	QIDS OASIS		Depression score: mean 10.74 OHC vs 8.39 no OHC, *t* = − 2.27 (*p* = .025) Anxiety score: mean 6.63 OHC vs 4.66 no OHC, *t* = − 1.90 (*p* = .060)
Côté et al. (2018), Finland	Prospective cohort *Linked national registers*	Child welfare population and matched controls	Adults with and without OHC history, matched on individual and family characteristics *F* = 49.0%	772	Any disorder** Psychotic/bipolar disorders Depression/anxiety disorders Comorbidity**	Psychiatric in/outpatient Prescribed psychotropic medication	Matched controls	Likelihood OHC vs no OHC: Any mental disorder OR = 2.36 (95% CI 1.68, 3.31) Psychotic/bipolar disorders OR = 3.98 (95% CI 1.80, 8.80) Depression/anxiety disorders OR = 2.15 (95% CI 1.46, 3.18) Comorbidity ≥ 3 diagnoses OR = 2.79 (95% CI 1.40, 5.41) Psychotropic medication OR = 1.96 (95% CI 1.38, 2.80)
Courtney et al. (2001), USA	Prospective cohort *Foster Youth Transitions to Adulthood Study*	Child welfare population	Young adults ≥ 18 months OHC with no developmental disability *F* = 57.0%	141	Psychological distress Service use**	MHI Self-report		Psychological distress: mean score 65 for Caucasians and 67 for African Americans Received mental health service 21%
Courtney et al.* (2007), USA	Prospective cohort *Midwest Study*	Child welfare population	Young adults placed in OHC < age 16 years due to abuse or neglect and who aged out of the welfare system *F* = 53.0%	590	Any disorder** PTSD Depression Anxiety Service use	CIDI Self-report		At age 21 years Any mental health disorder: *F* = 14.2% *M* = 4.6% PTSD: *F* = 7.9% *M* = 3.8% Major depression: *F* = 7.6% *M* = 1.1% Dysthymia: *F* = 0% *M* = 0% Generalised anxiety disorder: *F* = 0% *M* = 0% Psychological/emotional counselling 10.5% (vs 7.3% general population ‘Add Health’ survey) Psychotropic medication 12.7% Hospitalised for m/h reason 12.5%
Courtney et al.* (2010), USA	Prospective cohort *Midwest Study*	Child welfare population	Young adults placed in OHC < age 16 years due to abuse or neglect who aged out of the child welfare system *F* = 53.5%	602	Service use	Self-report		At age 23/24 years Psychological/emotional counselling 11.3% (vs 6.5% general population ‘Add Health’ survey) Psychotropic medication 11.8% Hospitalised for m/h reason 6.5%
Courtney et al.* (2011), USA	Prospective cohort *Midwest Study*	Child welfare population	Young adults placed in OHC < age 16 years due to abuse or neglect who aged out of the child welfare system *F* = 55.7%	596	Depression Suicide ideation Suicide attempt Social phobia PTSD Service use	CIDI Self-report		At age 26 years Depression symptoms (depressed for most of the day ≥ 2 weeks): *F* = 27.7% *M* = 19.0% Suicide ideation: *F* = 7.0% *M* = 4.9% Suicide attempt: *F* = 2.7% *M* = 1.5% Any PTSD symptom: *F* = 58.6% *M* = 56.4% Social phobia (at least one unusually strong symptom): *F* = 39.2% *M* = 31.2% Psychological/emotional counselling: *F* = 14.5% *M* = 8.8% Psychotropic medication *F* = 18.7% *M* = 9.1% Hospitalised for m/h reason *F* = 6.6% *M* = 8.0%
Dixon et al.* (2006), UK	Prospective cohort *Survey*	Child welfare population	Young people who had left OHC in 7 local authorities in England *F* = 53.0%	106	Mental health problems Suicide attempt	GHQ-12 Self-report	Demographics Care experiences Other	Mental health problems: 12% (3 months post OHC) and 24% (12 months post OHC) Suicide attempt: at least 4 young people 3–12 months post OHC OHC predictors of mental health problems (model not shown): High score for range of trouble while in OHC: *b* = 0.236 (*p* = 0.02) Duration in care (*ns*) Placement movement (*ns*) Age at leaving OHC (*ns*)
Dregan et al. (2011), UK	Prospective cohort *1970 British Cohort Study*	Birth cohort (1970)	Adults with and without OHC history *F* = nr	10,961	Depression	Malaise Inventory	Demographics Socio-economic Other	Likelihood of depression OHC vs no OHC: OR = 1.73 (95% CI 1.34, 2.24)
Garcia et al. (2012), USA	Cross-sectional *Casey National Alumni Study*	Child welfare population	Latino, Caucasian and African American adults ≥ 1 year Casey OHC *F* = 49.5%	805	At least 1 disorder**	CIDI	Demographics Care experiences	OHC likelihood at least one psychiatric disorder: Placement instability (Caucasians): OR = 1.40 (95% CI 1.10, 1.78) Circumstances of exit (*ns)* Drug/alcohol services (Caucasians): OR = 1.72 (95% CI 1.01, 2.93) Mental health services (*ns)* Tutoring (*ns)* Employment services (African Americans) OR = 10.85 (95% CI 2.32, 50.76) Independent living services (African Americans) OR = .165 (95% CI .050, 0.548) Tangible items for leaving (*ns*) Agency helpfulness (Caucasians): OR = 2.29 (95% CI 1.03, 5.07) Prepared for leaving (Caucasians): OR = .630 (95% CI 0.452, 0.878) Satisfaction with care (African Americans): OR = 0.660 (95% CI 0.487, 0.895)
Garcia et al. (2015), USA	Cross-sectional *Casey National Alumni Study*	Child welfare population	Latino, Caucasian and African American adults ≥ 1 year Casey OHC *F* = 49.5%	805	At least 1 disorder**	CIDI	ACEs Demographics Care experiences Other	OHC predictors at least one psychiatric disorder: Placement instability: *b* = 0.366 (*p* < 0.001) Agency helpfulness: African American *b* = − 0.984 (*p* < 0.01); Caucasian *b* = − 0.395 (*p* < 0.01)
Harris et al. (2010), USA	Cross-sectional *Casey National Alumni Study*	Child welfare population	Caucasian and African American adults ≥ 1 year Casey OHC *F* = 52.6% (African American) *F* = 48.2% (Caucasian)	708	Overall mental health At least 1 disorder** ≥ 3 disorders** Depression Panic syndrome Modified Social Phobia Anxiety PTSD	SF-12 CIDI	Demographics Care experiences	In bivariate analysis Modified Social Phobia (MSP) was the only outcome that showed statistically significant differences in mental health diagnosis between ethnic groups. In logistic regression (model not shown) ethnicity was not significantly associated with no MSP among OHC adults after controlling for demographic factors
Kessler et al. (2008), USA	Cross-sectional *Northwest Study*	Child welfare population	Adults ≥ 1 year in Casey or public OHC as adolescents *F* = 55.4% (private) *F* = 61.9% (public)	479	Depression Anxiety No. of mental disorders**	CIDI		Major depression: 11.3% private OHC vs 24.3% public OHC Anxiety disorders: 28.8% private OHC vs 43.0% public OHC No. of disorders: private OHC respondents had 44.7 fewer 12-month disorders per 100 individuals than public OHC
Patterson et al. (2015), Canada	Cross-sectional *Baseline data RCT*	Child welfare population and a comparison sample	Homeless/ precariously housed adults with a current mental disorder, with and without OHC history *F* = 28.0%	442	Severe cluster Less severe cluster Multiple disorders Depression Panic disorder PTSD Hypo (manic) episode Mood disorder/psychotic Psychotic disorder High suicide risk	MINI	Demographics Socio-economic	Likelihood OHC vs no OHC: Less severe cluster of mental disorders: OR = 1.62 (95% CI 1.03, 2.57) ≥ 2 mental disorders: OR = 1.69 (95% CI 1.08, 2.66) PTSD: OR = 1.60 (95% CI 0.98, 2.62) High suicide risk: OR 1.59 (95% CI 0.92, 2.72) In unadjusted models, major depressive episode, panic disorder, hypo manic episode, mood disorder with psychotic features, psychotic disorder, severe cluster were not significantly associated (*p* = < .0.05) with OHC history
Pecora et al.* (2005), USA	Cross-sectional *Northwest Study*	Child welfare population	Adults ≥ 12 months in Casey or public OHC as adolescents *F* = 60.5%	479	At least 1 diagnosis** ≥ 3 diagnoses** Depression Panic syndrome Modified social phobia Anxiety PTSD Overall mental health	CIDI SF-12		OHC vs general population (using National Comorbidity Study Replication data matched on age): At least one diagnosis 54.4% vs 22.1% (*p* = < 0.05) ≥ 3 diagnoses 19.9% vs 2.9% (*p* = < 0.05) PTSD 25.2% vs 4.0% (*p* = < 0.05) Major depression 20.1% vs 10.2% (*p* = <0.05) Panic syndrome 14.8% vs 3.5% (*p* = < 0.05) Modified social phobia 17.1% vs 8.8% (*p* = < 0.05) Generalised anxiety 11.5% vs 3.2% (*p* = < 0.05) SF-12 ≥ 50 good mental health 50.6% Statistical simulation used to optimise OHC experiences. Optimising placement history/experience and education services/experience reduced the number of poor mental health outcomes by 22.0% and 13.0% respectively
Power et al. (2002), UK	Prospective cohort *National Child Development Study*	Birth cohort (1958)	Adults with and without childhood OHC *F* = 57.5%	5340	Psychological distress	Malaise Inventory		Likelihood psychological distress, unskilled/manual vs professional/managerial classes: *M*: OR = 3.30. Adjusted for in care ever by age 16 OR = 2.90 (95% CI excludes 1) *F*: OR = 3.41. Adjusted for in care ever by age 16 OR = 3.31 (95% CI excludes 1)
Rebbe et al. (2017), USA	Prospective cohort *Midwest Study*	Child welfare population	Adults ≥ 1 year OHC who aged out of the child welfare system *F* = 51.6%	732	Depressive symptoms PTSD symptoms Service use	CIDI Self-report	Latent class analysis	In latent class analysis three classes were identified: Complex Adversity, Environmental Adversity and Lower Adversity. The Complex Adversity group experienced significantly higher depressive symptoms, PTSD symptoms and mental health service use than other classes
Roller White et al. (2009), USA	Cross-sectional *Northwest Study*	Child welfare population	Adults ≥ 12 months in Casey or public OHC as adolescents *F* = 60.5%	479	Depression	CIDI	ACEs Demographics Care experiences Other	OHC likelihood *no* current depression: No. placements low vs high: OR = 1.8 (*p* = < 0.05) Years in care low vs high: OR = 1.3 (*p* = *ns*) Placement change rate low vs high: OR = 1.7 (*p* = < 0.05) No. reunification failures low vs high: OR = 1.3 (*p* = *ns*) No of runaways low vs high: OR = 1.5 (*p* = < 0.05) No. of unlicensed living with friend/relative low vs high: OR = 3.5 (*p* = < 0.05) No. school changes low vs high: OR = 1.7 (*p* = < 0.05) Accessed tutoring/other education services: OR = 1.8 (*p* = < 0.05) Accessed therapeutic services: OR = 2.5 (*p* = < 0.05) Fun & religious activities with foster family: OR = 1.4 (*p* = < 0.05) Preparation for leaving OHC high vs low: OR = 2.6 (*p* = < 0.05) Leaving OHC resources high vs low: OR = 2.1 (*p* = < 0.05) Positive parenting during OHC high vs low: OR = 1.4 (*p* = *ns*) Felt loved: OR = 2.0 (*p* = <0 .05) Foster parents helpful very vs a little: OR = 2.5 (*p* = < 0.05) Close relationship with an adult: OR = 0.7 (p = < 0.05) Received help with ethnic identity: OR = 1.6 (*p* = < 0.05) No maltreatment during OHC: OR = 2.7 (*p* = < 0.05) Physical abuse only vs sexual abuse and other maltreatment during OHC: OR = 6.1 (*p* = < 0.05)
Roller White et al. (2015), USA	Cross-sectional *The Michigan Alumni Study*	Child welfare population	Adults ≥ 1 year OHC placed < 16 years due to child maltreatment and/or child behaviour problems *F* = 66.2%	65	At least 1 diagnosis** ≥ 3 diagnoses** Depression Anxiety PTSD Social phobia Mania Panic disorder	CIDI		OHC vs general population (using National Comorbidity Survey Replication matched on age, ethnicity and gender) At least one diagnosis 50.8% vs 36.3% (*p* = < 0.05) ≥ 3 more diagnoses 10.8% vs 10.4% (*p* = *ns*) Depression 10.8% vs 16.4% (*p* = *ns*) Generalised anxiety 4.6% vs 9.5% (*p* = *ns*) Mania 12.3% vs 1.0% (*p* = < 0.05) Panic disorder 1.5% vs 8.6% (*p* = < 0.05) PTSD 23.1% vs 7.5% (*p* = < 0.05) Social phobia 16.9% vs 10.8% (*p* = *ns*)
Roos et al. (2014), Canada	Cross-sectional *Baseline data RCT*	Child welfare population and a comparison sample	Homeless/ precariously housed adults with a current mental disorder, with and without a childhood history of OHC *F* = 36.5%	504	Depression Manic/hypomanic Panic disorder PTSD Mood disorder/psychotic Psychotic disorder Suicidality Comorbidity**	MINI SF-12 CIDI	Demographics Socio-economic	Likelihood OHC vs no OHC: Major depressive episode OR = 1.53 (95% CI 0.95, 2.46) Manic/hypomanic OR = 0.97 (95% CI 0.58, 1.62) Panic disorder OR = 0.83 (95% CI 0.55, 1.24) PTSD OR = 0.81 (95% CI 0.53, 1.23) Mood disorder/psychotic OR = 1.02 (95% CI 0.64, 1.63) Psychotic disorder OR = 0.57 (95% CI 0.37, 0.88) Suicidality OR = 1.13 (95% CI 0.76, 1.71) Comorbidity > 3 OR = 0.943 (95% CI 0.61, 1.40)
Rutman et al.* (2007), Canada	Prospective cohort *Promoting Positive Outcomes for Youth from Care*	Child welfare population	Young adults with a history of OHC followed up 6–9 month intervals after leaving OHC *F* = 78.4%	37	Depression Mental health condition**	Self-report		At T2: 48.0% either reported experiencing depression or concerns and/or treatment related to depression At T3: 44.0% either reported having depression or concerns and/or treatment related to depression T4: 43.0% reported depression or concerns and/or treatment related to depression. Presence of a mental health condition: 51.0% (T2); 44.0% (T3); 57.0% (T4)
Schneider et al. (2009), USA	Cross-sectional *California Women's Health Survey (2001–2004)*	Child welfare population and a comparison sample	Women with and without a history of OHC *F* = 100.0%	9,608	Mental distress PTSD	HRQOL PC-PTSD	Demographics	Likelihood OHC vs no OHC: Frequent mental distress OR = 1.43 (95% CI 1.10, 1.84) Probable PTSD OR = 2.85 (95% CI 2.15,3.79)
Smith* (2017), UK	Cross-sectional S*urvey of Barnardo's Leaving Care Workers*	Child welfare population	Young care leavers supported by Barnardo’s *F* = nr	274	Mental health problems	Service case files		Mental health needs 46.0% Mental health crisis since leaving care 25.0%
Teyhan et al. (2018), UK	Prospective cohort *Avon Longitudinal Study of Parents and Children*	Child welfare population and a comparison sample	Pregnant women and their male partners with and without a history of childhood OHC *F* = 70.6%	12,429	Depression Anxiety	Edinburgh Postnatal Depression Scale Anxiety subscale, Crown-Crisp Experiential Index	Demographics Socio-economic Other	Likelihood OHC vs no OHC: High depression score during pregnancy M: OR = 2.3 (95% CI 1.1, 4.5) F: OR = 2.0 (95% CI 1.3, 3.1) High anxiety score during pregnancy M: OR = 1.6 (95% CI 0.7, 3.7) F: OR = 2.0 (95% CI 1.3, 3.1) When child age 5 years old: High anxiety score F: OR = 1.5 (95% CI 1.0, 2.5) High depression score F: OR = 1.6 (95% CI 1.0, 2.5) Anxiety symptoms past year M: OR = 1.0 (95% CI 0.5, 2.2) F: OR = 1.4 (95% CI 1.0, 2.1) Depression symptoms past year M: OR = 1.0 (95% CI 0.4, 2.2) F: OR = 1.6 (95% CI 1.1, 2.3)
Villegas and Pecora (2012), USA	Cross-sectional *Casey National Alumni Study*	Child welfare population	Hispanic, African American and White adults in Casey OHC ≥ 1 year *F* = 55.4%	810	At least 1 diagnosis** Overall mental health Successful mental health	CIDI SF-12 CIDI and SF-12 combined	ACEs Demographics Care experiences	OHC likelihood of successful mental health: Placed age 0-5 years vs ≥ age 12: OR = 1.759 (*p* = 0.006) Maltreatment in OHC: OR = 0.683 (*p* = 0.031) No. placements < 4 vs ≥ 9: OR = 1.646 (*p* = 0.015) Prep. for leaving low vs high: OR = 0.531 (*p* = 0.009)
Viner and Taylor (2005), UK	Prospective cohort *1970 British Birth Cohort*	Birth cohort (1970)	Adults with and without a childhood history of OHC *F* = nr	9,557	Psychological morbidity Service use**	Malaise Inventory Self-report	Socio-economic	Likelihood psychological morbidity OHC vs no OHC: M: OR = 1.8 (95% CI 1.1, 3.0), F: OR = 1.6 (95% CI 1.1, 2.3) Likelihood seen specialist OHC vs no OHC: M: OR = 1.7 (95% CI 1.1, 2.6), F: OR = 1.1 (95% CI 0.7, 1.5)
Vinnerljung and Hjern (2014), Sweden	Prospective cohort *Linked national registers*	Birth cohorts (1973–1981)	Adults with a history of OHC ≥ 6 months (excluding respite care) and general population peers. OHC categorised 1) short/med 0.5–5 years 2) long-term > 5 years 3) teen placements *F* = 48.0%	765,038	Mental health problems	Prescribed psychotropic medication	ACEs Demographics Socio-economic	Risk at least one prescribed psychotropic drug OHC vs no OHC: Short/med M: HR = 1.88 (95% CI 1.67, 2.11), F: HR = 1.51 (95% CI 1.36, 1.69) Long-term M: HR = 1.52 (95% CI 1.36, 1.71), F: HR = 1.45 (95% CI 1.32, 1.60) Teen placed M: HR = 2.73 (95% CI 2.57, 2.91), F: HR = 2.26 (95% CI 2.14, 2.38) HRs were significantly higher for all OHC groups compared to majority peers for neuroleptics, antidepressants and anxiolytics/hypnotics
Wall-Wieler et al. (2018), Sweden	Prospective cohort *Linked national registers*	Birth cohorts (1973–1980)	Adult parents who spent time in OHC and parents with no OHC *F* = 53.1%	487,948	Suicide mortality	Death records	Demographics Socio-economic Other	Risk death by suicide OHC vs no OHC Mothers in OHC as a child: HR = 2.35 (95% CI 1.27, 4.35) Fathers in OHC as a child: HR = 1.06 (95% CI 0.69, 1.63)
Zlotnick et al. (2012), USA	Cross-sectional *California Health Interview Survey* *(2003 and 2005)*	Child welfare population and a comparison sample	Adults with and without a childhood history of OHC *F* = 50.8%	70,456	Mental health problems	Self-report	Demographics Socio-economic	Likelihood mental health problems OHC vs no OHC: Total sample: OR = 1.62 (95% CI 1.42, 1.85) 18–25 years: OR = 1.41 (95% CI 1.02, 1.94) 26–35 years: OR = 1.32 (95% CI 0.98, 1.77) 36–45 years: OR = 1.64 (95% CI 1.23, 2.20) 46–55 years: OR = 2.10 (95% CI 1.60, 2.76) 56–65 years: OR = 1.47 (95% CI 1.11, 1.95) > 65 years: OR = 1.64 (95% CI 1.18, 2.29)

*ACE* Adverse Childhood Experience, *ns*  not significant, *nr*  not reported, * SF-12*  Short Form Health Survey, *CIDI* Composite International Diagnostic Interview; *GHQ* General Health Questionnaire, *Rutter ‘A’* Rutter 'A' Health and Behaviour Checklist, *QIDS*  Quick Inventory of Depressive Symptomatology, *OASIS* Overall Anxiety Severity and Impairment Scale, *MHI* Mental Health Inventory, *MINI* MINI International Neuropsychiatric Interview; *HRQOL-14;CDC* Health Related Quality of Life 14 item measure, Centres for Disease Control and Prevention, *PC-PTSD* Primary Care PTSD Screen

*Report

**Aggregate measure may include drug/alcohol/eating or other disorders

†Full details of individual covariates utilised in regression models are available in online supplementary Table TS2

Adult mental health outcomes were defined using six different measures/indicators: psychometric screening instruments (33/55), self-report (14/55), hospital records (9/55), psychotropic medication uptake (7/55); death records (3/55) and service case files (1/55). Fifteen different psychometric screening instruments were used to assess mental health status. To identify depression alone, six different screening instruments were used. A wide range of mental health outcomes have been examined (Supplementary figure FS1). Outcomes are reported as defined by individual studies, but definitions were not always consistent. For example, studies using the Rutter Malaise Inventory [38] to measure mental health have defined the outcome variously as ‘depression’ [39, 40], ‘depression tendency’ [41, 42], ‘psychological morbidity’ [43] or ‘psychological distress’ [44]. Outcome timeframes for assessing mental health status varied widely and included current status, past few days or weeks, past year or at a specific age or age range. Twenty-six studies examined a single mental health outcome and twenty-nine examined two or more. The ‘mental ill-health’ category (40/55) includes general psychological morbidity or any unspecified mental disorder. Depression was the most studied specific mental health condition (27/55). Depression studies primarily used psychometric screening tools (20/27), though self-report measures (4/27) and hospital/medication records were also used (3/27). Similarly, anxiety (12/55), post-traumatic stress disorder (PTSD) (13/55), psychotic/bipolar disorders (8/55) and the presence of multiple comorbid mental health disorders (8/55) were assessed most frequently using screening instruments. Panic disorder (7/55) and social phobia (6/55) were measured using screening instruments only, primarily the Composite International Diagnostic Interview (CIDI) [45]. Suicide attempt (7/55) was measured most frequently using hospital records. Mental health service use (7/55) was measured using self-report only. Overall suicide ideation (5/55) and suicide deaths (3/55) as mental health outcomes have received the least attention.

The review identified four main areas within the research on child welfare service involvement and adult mental health outcomes based broadly on service type: (1) any OHC (35/55); (2) OHC placement types (residential, foster, kinship) (11/55); (3) both IHC and OHC (4/55) and (4) IHC only (5/55). Terminology can be confusing as in the USA the term ‘foster care’ is synonymous with OHC, including what is understood elsewhere as distinct placement types (residential, foster care by non-relatives or family) and the term ‘family foster care’ equates to what is understood as non-kinship foster care in other countries [2]. Terminology used to describe statutory placement outside the home included ‘public care’, ‘out-of-home-care’, ‘societal care’, ‘looked after’ and ‘in care’. Results of the narrative synthesis within each of these areas are presented below.

## Out-of-home care

Thirty-five studies examined the relationship between experience of OHC and adult mental health without differentiating outcomes by OHC placement type (Table 1). Of these, 18/35 were conducted in the USA, 9/35 in the UK, 4/35 in Sweden, 3/35 in Canada and one in Finland. In this category, 11/35 studies reported prevalence data [39, 46–55], 14/35 examined mental health using regression models comparing adults with a history of OHC to adults in the general population [40–43, 56–65], 9/35 investigated risk factors within OHC populations [66–74] and one identified care history as a risk factor for class differences in adult psychological distress [44].

### Prevalence

Prevalence rates for specific mental health conditions varied widely by study. The majority reported prevalence of mental health conditions and mental health service use as higher among adults with a history of OHC compared to adults with no care history [40–43, 46, 47, 49–51, 53, 56, 57, 61, 62, 64, 65] though some also reported higher rates of mental ill-health among non-OHC groups for certain diagnoses or sub-groups [39, 47, 56, 64]. Prevalence of mental ill-health was generally higher among care experienced women than men [41, 43, 49, 51, 56, 57, 60] though this pattern was not universal. A minority of studies reported mixed findings with elevated rates among men at some time points [41, 42, 56]. The incidence of suicide mortality was higher among men than women with OHC history [58].

### Studies of OHC compared to the general population

Fourteen of the thirty-five OHC studies used statistical models to test whether placement in OHC was associated with adult mental health using no care history as the comparator. Of these, six studies reported that OHC was associated with adverse adult mental health outcomes including psychological distress, specific disorders and comorbid mental health disorders [40, 57, 60–62, 65]. The remaining eight studies found associations differed according to gender, age and diagnostic sub-category. Two studies based on the UK 1958 birth cohort found OHC during childhood was significantly associated with depression tendency in men but not women at age 33 years [41, 42]. A study based on the UK 1970 birth cohort found care history was associated with psychological morbidity in both genders though only men were more likely to have seen a mental health specialist [43]. Another UK study of pregnant women and their partners found women but not men with OHC history had higher odds of experiencing anxiety and depression when the child was 5 years old compared to those without care experience [56]. A Swedish study found that mothers, but not fathers, who had spent time in care had an increased risk of death by suicide [58]. A USA study showed that OHC history was associated with likelihood of reporting a mental health problem in the last 30 days in a cohort of individuals aged 18–65 years, but when stratified by age the association did not remain significant in the 26–35 years age group [59]. Two Canadian studies using samples of homeless adults experiencing current mental illness found mixed associations depending on the disorder(s) examined [63, 64].

Among these 14 studies, there was considerable heterogeneity in the number and range of covariates included in the analyses. Only three studies accounted for parental mental-ill health or substance misuse and no study accounted for childhood maltreatment [57, 60, 62]. One study explored care experiences by categorising OHC adults according to the time spent in care and age at first placement, finding that individuals placed as teenagers faced the highest risk for mental health problems. Risks were slightly lower for adults who experienced a long-term placement than those with a short to medium-term stay [57].

### ‘Within group’ studies of OHC

Nine studies in this category comprised OHC populations without a ‘no care’ control group and examined the risk factors for poor mental health outcomes within OHC experiences. Findings on the significance of experiences during care were often mixed. For example, placement instability was significantly associated with adult mental-ill health in five studies [66–69, 74] but not in two studies [71, 73]. Rebbe et al. used Latent Class Analysis to identify three distinct classes of OHC experience [72]. The class characterised by a higher number of placements experienced significantly higher depressive symptoms, PTSD symptoms and mental health service use than the other classes. Villegas et al. found younger age at placement in OHC was associated with improved mental health outcomes [66], whereas Anctil et al. observed that age at placement was not a significant risk factor for adults with a history of OHC who had been diagnosed during childhood with a physical or psychiatric impairment [69]*.* Duration in OHC was not significantly associated with mental health outcomes [73, 74]*.* The degree of help/support received and preparation for leaving care affected likelihood of poor outcomes [66–68, 74]. Experiencing maltreatment during OHC was associated with adverse mental health in adulthood [66, 74]. Studies reported ethnicity was not associated with mental health outcomes among adults with OHC history [66, 69, 70, 73], however care experiences operated differently by ethnic group [67, 68]. For example, placement instability was associated with increased likelihood of psychiatric diagnosis for Caucasians but not African Americans [67]. In this group, five studies accounted for pre-care maltreatment when exploring adult mental health outcomes [68, 69, 71, 72, 74].

## Out-of-home care placement type

Eleven studies examined the relationship between specific OHC placement type (kinship, foster or residential care) and adult mental health (Table 2). Of these, 7/11 were conducted in the USA [75–81], 2/11 in the UK [82, 83] and 2/11 in Sweden [84, 85].

**Table 2:** Included studies of OHC placement type and adult mental health

Study	Design *Data source*	Sample	Sample description	*N*	Mental health outcome	Measure	Covariates†	Summary of main results
Benedict et al. (1996), USA	Cross-sectional *Survey and case records*	Child welfare population	Adults with a history of kinship (> 50% OHC in kinship) and other OHC *F* = 55.0%	214	Mental health problems	GHQ-28	Demographics Care experiences Other	OHC likelihood higher no. of mental health problems: Kinship vs other OHC: Log odds = 1.34 (95% CI 0.70, 2.56) Maltreatment in OHC: Log odds = 5.59 (95% CI 1.96, 15.99) Maternal drug use + kinship: Log odds = 3.58 (95% CI 1.34, 9.50) Mental health problems in OHC: Log odds = 2.10 (95% CI 1.16, 3.81) Adoption vs other outcome: Log odds = 4.42 (95% CI 1.16, 16.83)
Buehler et al. (2000), USA	Cross-sectional *National Survey of Families and Households (1988)*	Child welfare population, matched controls and a comparison sample	Adults ≥ 6 months non-kinship foster care, matched controls and general comparison group *F* = 63.0%	303	Depression	Self-report	Matched controls	Depressive affect mean score: Non-kinship foster = 1.78 Matched group = 1.41 General population = 1.45 Difference between groups (*ns)*
Carpenter and Clyman (2004), USA	Cross-sectional *National Survey of Family Growth* (*1995)*	Child welfare population and a comparison sample	Women ≥ 1 month kinship care only and women who lived with at least one biological or adoptive parent throughout childhood *F* = 100.0%	8,760	Anxiety	Self-report	ACEs Demographics Socio-economic	Likelihood anxiety kinship vs no OHC: OR = 1.6 (95% CI 1.1, 2.2)
Cook-Fong (2000), USA	Cross-sectional *National Survey of Families and Households (1988)*	Child welfare population and a comparison sample	Adults with a history of non-kinship foster care and general population *F* = nr	13,017	Depression	Self-report	Demographics Socio-economic	Non-kinship foster vs no OHC predicted higher depression score: b = 3.418 (*p* < 0.05)
Dregan and Gulliford (2012), UK	Prospective cohort *1970 British Cohort Study*	Birth cohort (1970)	Adults with childhood history of foster care, or residential care, or both, and general population *F* = 51.0%	10,895	Depression	Malaise Inventory	ACEs Demographics Socio-economic Care experiences Other	Likelihood depression OHC vs no OHC: Residential care OR = 1.81 (95% CI 1.23, 2.68) Foster care OR = 1.39 (95% CI 0.90, 2.16) Both residential and foster OR = 1.91 (95% CI 0.98, 3.73) Placed < 1 year old OR = 1.54 (95% CI 0.92, 2.52) Placed age 1–4 years OR = 1.23 (95% CI 0.68, 2.21) Placed age 5–10 years OR = 1.66 (95% CI 0.91, 3.02) Placed > age 10 years OR = 1.41 (95% CI 0.74, 2.68) Duration < 3 months OR = 1.79 (95% CI 1.10, 2.89) Duration 4–12 months OR = 2.27 (95% CI 1.31, 3.93) Duration > 1 year OR = 2.05 (95% CI 1.33, 3.14) 1 placement OR = 1.97 (95% CI 1.24, 3.14) ≥ 2 placements OR = 1.86 (95% CI 1.19, 2.91)
Fechter-Leggett and O'Brien (2010), USA	Cross-sectional *Casey National Alumni Study*	Child welfare population	Adults in Casey OHC ≥ 1 year categorised according to time spent in kinship care/other OHC *F* = 47.9%	1,068	Any diagnosis** ≥ 3 diagnoses** Depression Panic syndrome Social phobia PTSD Anxiety Overall mental health	CIDI SF-12	ACEs Demographics Care experiences Other	100% kinship vs other placement patterns was not associated with any mental health outcome. Care factors operated differently according to mental health outcome examined. For example, longer duration in OHC increased the likelihood of ‘any’ diagnosis (≥ 10 years OHC vs < 6 years: OR = 1.4, *p* = < 0.05) but was not associated with other mental health outcomes
Hjern et al. (2018), Sweden	Prospective cohort *Linked national registers*	Birth cohorts (1972–1981)	Adults with a childhood history of long-term foster care (≥ 10 years) and general population F = 50.6% (foster) F = 48.6% (general)	941,455	Depression	Prescribed antidepressants Psychiatric in/outpatient	Demographics Socio-economic	Risk of antidepressant medication foster care vs no OHC: HR = 1.44 (95% CI 1.29, 1.61) Risk of hospital psychiatric care foster care vs no OHC HR = 1.62 (95% CI 1.35, 1.94)
Jackson et al. (2011), USA	Cross-sectional *Casey National Alumni Study*	Child welfare population	African American and White Adults in Casey OHC ≥ 1 year categorised as spending any time in kinship care or other OHC *F* = 49.2%	708	PTSD	CIDI	ACEs Demographics Socio-economic Care experiences Other	OHC likelihood PTSD: Kinship care vs other OHC: OR = 2.58 (95% CI 1.26, 5.28) Kinship care and female: OR = 0.31 (95% CI 0.13, 0.77) Placement change rate medium vs low: OR = 1.03 (95% CI 0.62, 1.72) Placement change rate high vs low: OR = 1.17 (95% CI 0.70, 1.94) Maltreated in OHC: OR = 1.64 (95% CI 1.08, 2.49)
Jackson Foster et al. (2015), USA	Cross-sectional *Casey National Alumni Study*	Child welfare population	Adults in Casey OHC ≥ 1 year categorised as spending any time in kinship care or other OHC *F* = nr	1038	Depression Anxiety Panic disorder PTSD Social phobia Comorbidity**	CIDI	Demographics Socio-economic Care experiences Other	OHC likelihood comorbidity: Kinship vs other OHC: OR = 0.97 (95% CI 0.7, 1.3) Felt loved by foster parents: OR = 0.86 (95% CI 0.6, 1.2) Close adult relationship: OR = 1.01 (95% CI 0.8, 1.3) Foster parents sometimes helpful vs little help: OR = 0.63 (95% CI 0.5, 0.9) Foster parents helpful a lot vs little help: OR = 0.68 (95% CI 0.5, 1.0) Maltreated in OHC: 1.73 (95% CI 1.3–2.3)
Pritchard and King (2000), UK	Prospective cohort *Police records and Suicide Register*	Child welfare population and a comparison sample	Adults in residential care or an Exclusion Unit at some point age 11–15 years *F* = 37.3%	1041	Suicide mortality	Suicide register		There were no male or female suicides in the residential care group
Vinnerljung and Sallnäs (2008), Sweden	Prospective cohort *Case files and linked national registers*	Child welfare population	Adults who entered residential or foster care aged 13–16 years *F* = 48.5%	718	Mental health problems**	Psychiatric inpatient	ACEs Demographics Care experiences	OHC likelihood hospitalisation for mental health reasons: Residential vs foster OHC: OR = 1.7 (95% CI 1.1, 2.7) Placement breakdown: OR = 1.8 (95% CI 1.2, 2.7) Duration (*ns)* Age placed (*ns)* Placed for behaviour reasons (*ns)*

*ACE*  Adverse Childhood Experience, *ns* not significant, *nr* not reported, * GHQ*  General Health Questionnaire, *SF-12* Short Form Health Survey, *CIDI*  Composite International Diagnostic Interview

**aggregate measure may include drug/alcohol/eating disorders; †Full details of individual covariates used in regression models are available in online supplementary Table TS3

### Studies of kinship foster care

Five of the seven USA studies examined the association between kinship care and adult mental health, though kinship experience and comparison groups were defined differently. Experience of kinship care only (i.e. no other OHC placement) was associated with increased likelihood of anxiety in women [77]. Spending more than 50% of OHC in kinship placement compared to non-kinship OHC was not associated with mental health [75]. Three studies used data from the Casey National Alumni Study in the USA, with mixed results. Any time in kinship care compared to other types of OHC was associated with increased likelihood of past-year PTSD, though this was moderated by gender which reduced the likelihood among women [80]. Any time in kinship care compared to other types of OHC was not significantly associated with the likelihood of comorbid psychiatric disorders [81]. Being placed only in kinship care compared to other placement patterns was not associated with any psychiatric disorder [79].

### Studies of non-kinship foster care

In this category, three studies examined the relationship between non-kinship foster care and depression. Using the 1998 National Survey of Families and Households, Cook-Fong found experience of non-kinship foster care compared to no OHC was associated with adult depression [78]. Using the same data source but different methods, Buehler et al. found a higher depression score among the foster group than matched controls but the differences were not statistically significant [76]. A Swedish study found long-term foster care (at least ten years) was associated with increased risk of depression [84]. It was not clear whether samples experienced other types of placement in addition to non-kinship foster care [76, 78, 84].

### Studies of residential care

Regarding residential care, a UK study found exclusive residential care (i.e. child not also placed in other types of care) was significantly associated with adult depression compared to no OHC experience [82]. Another UK study found no incidence of suicide among adults with experience of residential care, though the sample was small [83]. In Sweden, individuals who had been placed in residential care as teenagers had higher odds of hospitalisation for mental health reasons than those placed in foster care [85].

While most studies in the OHC placement type category controlled for basic demographics, the inclusion of pre-care adversity or adult socio-economic covariates was limited. Of the eleven studies, four accounted for pre-care maltreatment [79, 80, 82, 85]. Using their full OHC samples (i.e. not specific placement types) 6/11 studies examined the relationship between a range of care experience factors and mental health outcomes. Findings were again mixed. In one USA study, a longer duration in OHC was associated with increased likelihood of ‘any’ CIDI diagnosis, yet duration was not a significant risk factor for depression, panic syndrome, social phobia, PTSD, anxiety, comorbid disorders or overall mental health [79]. In contrast, a UK study found a longer duration in OHC was associated with increased likelihood of depression although it is worth noting the comparison group was adults with no OHC experience [82]. In Sweden, duration in OHC as a teenager was not significantly associated with mental health problems as indicated by psychiatric hospitalisation [85]. Regarding placement stability, results were again inconsistent between studies. Placement stability, defined as placement change rate, was not associated with PTSD in adults with OHC history [80]. Elsewhere, depression outcomes appeared similar for individuals placed in OHC on just one occasion or two or more occasions compared to adults with no OHC history [82]. Breakdown of a placement was associated with increased odds of psychiatric hospital admission [85]. In this category of studies, age at entry to OHC was not significantly associated with any mental health outcomes [82, 85]. Concerning gender, prevalence was higher among women than men for depression and PTSD [80, 84]. One study found rates of psychiatric hospitalisation were higher among men [85].

## Both in-home and out-of-home care

Only four studies have examined adult mental health outcomes using both OHC and IHC concurrent samples and all were conducted in Sweden utilising linked national registers (Table 3). Children may experience OHC following IHC, or repeat episodes of placement and reunification under supervision. The use of concurrent samples allows comparison based on highest level of exposure. One study found prevalence of suicide attempt and psychiatric hospital admission was generally higher in adults in receipt of disability pension with a history of OHC than those with a history of IHC, but psychotropic medication use was higher in those with a history of IHC compared to some OHC sub-groups [86]. Compared to adults in the general population, both IHC and OHC resulted in elevated risk for adult psychiatric disorder, suicide attempt and suicide mortality [87–89]. Risks for OHC adults were higher than IHC adults for suicide attempt [87] but broadly similar for suicide mortality [89], compared to peers with no child welfare experience. When stratified by gender, the risk for suicide mortality among IHC males was higher than for OHC males compared the general population. Duration in OHC was a significant risk factor for psychiatric disorder [88]. Only one study directly compared risk between OHC and IHC groups [89]. Risk for suicide mortality was higher among OHC women than IHC women, but the reverse for men [89]. The differences in risk ratios between OHC and IHC, however, were not statistically significant. While two studies included parental psychiatric disorders or substance misuse as covariates [87, 88], none controlled for childhood maltreatment.

**Table 3:** Included studies of both OHC and IHC and adult mental health

Study	Design *Data source*	Sample	Sample description	*N*	Mental health outcome	Measure	Covariates†	Summary of main results
Berlin et al. (2011), Sweden	Prospective cohort *Linked national registers*	Birth cohorts (1972–1981)	Adults ≥ 5 years OHC, adults who had IHC before their teens and general population *F* = 46.0% (OHC) *F* = 44.0% (IHC)	913,207	Suicide attempt	Psychiatric inpatient	ACEs Demographics Socio-economic	Relative Risk suicide attempt vs general population IHC: RR = 1.66 (95% CI 1.42, 1.95) OHC: RR = 2.28 (95% CI 1.97, 2.65)
Vinnerljung and Ribe (2001), Sweden	Prospective cohort *Linked national registers*	Birth cohorts (1969–1976)	Adults placed in OHC < age 13 years, adults with IHC history (Child Welfare Board involvement, a Contact Person assigned or residential care before age 7 but no foster care before age 13) and general population *F* = 47.2% (OHC) *F* = 45.4% (IHC)	13,100 (OHC) 10,668 (IHC)	Suicide mortality	Death records	Demographics Other	Risk Ratio suicide mortality OHC vs general population: All genders RR = 2.45 (95% CI 1.48, 3.42) M: RR = 2.31 (95% CI 1.21, 3.41) F: RR = 2.82 (95% CI 0.82, 4.82) Risk Ratio suicide mortality IHC vs general population: All genders RR = 2.44 (95% CI 1.46, 3.42) M: RR = 2.76 (95% CI 1.55, 3.97) F: RR = 1.52 (95% CI 0.00, 3.04) Difference in RR between IHC and OHC (*ns)*
Vinnerljung et al. (2006), Sweden	Prospective cohort *Linked national registers*	Birth cohorts (1973–1982)	Adults placed in OHC < age13 years, adults with IHC history (Contact Family) and general population. OHC categorised as: short-term (0–24 months, mix of residential/foster); intermediate (25–60 months, mostly foster); long-term (> 60 months, almost all foster) *F* = 48.8%	989,871	Suicide attempt Any psychiatric disorder	Psychiatric inpatient	ACEs Demographics Socio-economic	Relative Risk suicide attempt vs general population IHC: RR = 1.9 (95% CI 1.5, 2.4) Short-term OHC: RR = 2.1 (95% CI 1.8, 2.5) Intermediate OHC: RR = 2.0 (95% CI 1.5, 2.7) Long-term OHC: RR = 2.3 (95% CI 1.8, 2.8) Relative Risk any psychiatric disorder vs general population IHC: RR = 2.4 (95% CI 1.7, 3.2) Short-term OHC: RR = 2.3 (95% CI 1.9, 2.8) Intermediate OHC: RR = 2.3 (95% CI 1.6, 3.3) Long-term OHC: RR = 2.9 (95% CI 2.2, 3.7)
Vinnerljung et al. (2015), Sweden	Prospective cohort *Linked national registers*	Birth cohorts (1973–1978)	Adults in receipt of a disability pension (DP) with OHC or IHC history, general population with and without DP OHC categorised as: short-term (1st placed < 13 years and < 2 years in OHC, *F* = 47.5%); intermediate (1st placed < 13 years and 2–5 years in OHC, *F* = 45.4%); long-term (1st placed < 13 years and > 5 years in OHC, *F* = 48.7%); teen placement: (1st placed at age 13 or older, *F* = 50.7%) IHC = receipt of respite care via Contact Family at any time before age 18 years no OHC, *F* = 40.0%	524, 882	Mental health problems Suicide attempt	Prescribed psychotropic medication Psychiatric in/outpatient		Psychotropic medication use: IHC *M* = 59.0% *F* = 58.4% Short OHC *M* = 53.2% *F* = 61.4% Intermediate OHC *M* = 66.0% *F* = 54.9% Long-term OHC *M* = 48.2% *F* = 61.9% Teen placed OHC *M* = 56.1% *F* = 66.5% Majority population with DP *M* = 58.0% *F* = 57.8% Psychiatric care: IHC *M* = 70.5% *F* = 58.4% Short OHC *M* = 72.3% *F* = 63.4% Intermediate OHC *M* = 86.8% *F* = 74.5% Long-term OHC *M* = 73.0% *F* = 75.5% Teen placed OHC *M* = 78.9% *F* = 84.3% Majority population with DP *M* = 63.9% *F* = 54.9% Suicide attempt: IHC *M* = 17.9% *F* = 20.5% Short OHC *M* = 23.4% *F* = 17.2% Intermediate OHC *M* = 18.9% *F* = 27.5% Long-term OHC *M* = 19.9% *F* = 26.5% Teen placed group M = 25.3% *F* = 36.1% Majority population with DP *M* = 11.8% *F* = 12.7%

*ACE * Adverse Childhood Experience, *ns*  non-significant

†Full details of individual covariates used in regression models are available in online supplementary Table TS4

## In-home care only

Five studies examined the relationship between IHC only and adult mental health, one in the Netherlands, one in Canada and three in Sweden (Table 4). IHC exposures examined varied from any contact with Child Protection Services (CPS) to differing degrees of investigation and service provision. The Dutch study used latent class analysis to identify four classes of multi-problem young adult males involved with CPS during childhood [90]. There were significant differences between classes on anxious/depressive problems based on the timing, level and frequency of CPS involvement during childhood. The Canadian study found no association between CPS contact and past year suicide ideation/plans/attempts, self-reported mental disorder, or psychological distress among adults who had experienced child abuse compared to abused adults without CPS contact [91]. In Sweden, the provision of child protection services at least once predicted hospitalisation for a psychiatric diagnosis or suicide attempt in adulthood compared to being investigated but not having received any service [92]. For adults that received services, the prevalence of suicide attempt and psychiatric diagnoses was higher among women than men [92]. Two studies estimated the impact of Sweden’s Contact Family Program, a routine welfare service where a volunteer family/adult external to the family provides support to a child experiencing a stressful or adverse situation [93, 94]. Using latent class analysis and propensity score matching, there was little difference in risk of being in the outcome class characterised predominately by poor mental health for adults who received the service compared to matched controls [93, 94]. In variable-centred analysis, addressing poor mental health in isolation rather than grouped with other outcomes, risk of poor mental health was significantly higher for adults exposed to IHC compared to matched controls [94]. Three studies accounted for childhood abuse or neglect [90–92], but only one included a range of adult socio-economic covariates [91].

**Table 4:** Included studies of IHC and adult mental health

Study	Design *Data source*	Sample	Sample description	*N*	Mental health outcome	Measure	Covariates†	Summary of main results
Afifi et al. (2018), Canada	Cross-sectional *Canadian Community Health Survey-Mental Health (2012)*	Child welfare population and a comparison sample	Adults with a childhood history of sexual or physical abuse or exposure to intimate partner violence, with and without a history of Child Protection Service (CPS) contact *F* = nr	23,395	Any disorder ** Suicide ideation Suicide plans Suicide attempt Overall mental health Psychological distress	Self-report MHC-SF KDS	ACEs Demographics Socio-economic	Likelihood CPS contact vs no CPS contact: Any self-reported current mental disorder: OR = 1.20 (95% CI 0.9, 1.7) Past year suicide ideation: OR = 1.18 (95% CI 0.8, 1.8) Past year suicide plans: OR = 1.16 (95% CI 0.6, 2.4) Past year suicide attempts: OR = 0.86 (95% CI 0.3, 2.3) Languishing & moderate vs flourishing mental health: OR = 1.21 (95% CI 0.9, 1.7) CPS contact predictor of psychological distress: *b* = 0.74 (95% CI − 0.04, 1.5)
Brännström et al. (2013), Sweden	Prospective cohort *Linked national registers*	Birth cohorts (1980–1990)	Adults aged 18–29 years in Sweden who started IHC (Contact Family Program) at age 2–5 compared to matched and unmatched controls *F* = nr	954,848	Poor mental health	Prescribed psychotropic medication	Latent class analysis Matched controls	Risk Ratio poor mental health outcome class IHC vs unmatched peers RR = 1.21 (95% CI 1.11, 1.32) Risk Ratio poor mental health outcome class IHC vs matched peers RR = 1.08 (95% CI 0.96, 1.22)
Brännström et al. (2015), Sweden	Prospective cohort *Linked national registers*	Birth cohorts (1973–1984)	Adults aged 24–35 years in Sweden who started IHC (Contact Family/Person Program) at age 10–13 compared to matched and unmatched controls *F* = nr	1,002,122	Poor mental health	Prescribed psychotropic medication	Latent class analysis Matched controls	Risk Ratio poor mental health (variable-centred analysis) IHC vs unmatched peers RR = 1.93 (95% CI 1.83, 2.03) Risk Ratio poor mental health (variable-centred analysis) IHC vs matched peers RR = 1.23 (95% CI 1.14, 1.33) Risk Ratio poor mental health outcome class IHC vs unmatched peers RR = 1.08 (95% CI 1.00, 1.17) Risk Ratio poor mental health outcome class IHC vs matched peers RR = 0.91 (95% CI 0.82, 1.01)
van Duin et al. (2017), Netherlands	Cross-sectional *Survey and case records*	Child welfare population	Multi-problem young adult males with a history of Child Protection Service (CPS) involvement *F* = 0.0%	390	Anxiousness Depression	ASR	Latent class analysis	In latent class analysis (LCA) four classes were identified. The early CPS/family investigation group had the highest anxiousness/depression mean score (75.8). Post hoc test showed significantly higher anxiousness/depression in the early CPS/ family investigation group versus early CPS/multiple investigation group (*p* = 0.022)
Vinnerljung et al. (2006), Sweden	Prospective cohort *Case files and linked national registers*	Birth cohorts (1968–1975)	Adults who received Child Protection Agency (CPA) services at home, adults investigated by CPA but received no services and adults with no known CPA contact *F* = 40.0% (CPA) *F* = 29.0% (CPA no service)	2,232	Psychiatric diagnosis** Suicide attempt	Psychiatric inpatient	ACEs Demographics Socio-economic Other	CPA services at least once vs no CPA service predicted hospitalisation for psychiatric diagnosis/suicide attempt *β* = 0.43 (*p* < 0.001)

*ACE* Adverse Childhood Experience, * MHC-SF* Mental Health Continuum-Short Form, *KDS* Kessler Distress Scale, *ASR* Adult Self Report

**Aggregate measure may include substance misuse/eating disorder

†Full details of individual covariates are available in online supplementary Table TS5

## Discussion

This paper provides the first scoping review of adult mental health outcomes following receipt of child welfare services. The majority of studies reported that adults with an experience of any child welfare service have higher rates of adverse adult mental health outcomes and a higher risk of suicide compared to adults who, as children, had no experience of child welfare services. Predominantly studies suggest significant gender differences in mental health outcomes with higher prevalence in women compared to men [43, 49, 57, 60, 80, 84, 87, 92], except for suicide mortality which appears higher in child welfare experienced men compared to women [58, 89].

The literature presents conflicting findings on the importance of placement duration, placement stability and age at entry. Some studies suggest a longer duration in OHC is associated with worse mental health outcomes [79, 82, 88], others find no association [73, 74, 85]. Some studies suggest a younger age of entry is associated with better mental health outcomes [57, 66] whilst other find no association [69, 82, 85]. Regarding placement instability some studies suggest an association with worse mental health outcomes [66–69, 72, 74, 85] and others no association [71, 73, 80]. The quality of covariates varies greatly between studies with some studies only presenting prevalence rates which do not help in developing an understanding of the factors associated with adult mental health outcomes in children with child welfare experience.

There is marked variation in the quality and size of studies published to date. Population-wide studies are restricted to Swedish and Finnish register-based studies; elsewhere sampling methods and sizes varied considerably. The majority of studies were undertaken in the USA. Cross-national differences in child welfare systems and populations mean that findings cannot be uncritically applied between countries. However, in concurrence with earlier reviews discussed in the Introduction, this synthesis showed that independent of geography, measurement method or decade of birth, adults who were exposed to child welfare service experienced high rates and risks relating to a range of mental disorders, psychological morbidity, suicide attempt and suicide mortality compared to adults with no child welfare exposure [20, 21]. All studies which used statistical models reported a positive association between receipt of child welfare services and adult mental-ill health, though results did not always achieve significance across sample sub-groups and diagnostic categories. Measures of significant association ranged from RR = 1.21 (95% CI 1.11–1.32) [93] to OR = 5.1 (*p* =  < 0.001) [42] compared to adults without child welfare experience. This synthesis also presents a more nuanced picture and highlights the need for the research to go further, to attempt to separate out the effects of the welfare services themselves from the underlying reason a child becomes known to child welfare services to understand which aspects of welfare service interaction are associated with positive or negative mental health outcomes.

This review has also highlighted the need for more standardised nomenclature around both child welfare provision and mental health. Mental ill-health was defined by studies as a generalised condition of distress and/or as specific psychiatric disorders and measured in heterogeneous ways, making comparison problematic. The variation in prevalence and risks of mental ill-health and associated factors identified by individual studies is potentially explained by differences in the definitions of mental-ill health and measurement methods. This is a problem common to many aspects of population mental health research. Receipt of child welfare services is also described differently across countries. Whilst terminology, such as social care, social services and child welfare services, is not likely to change, it would be useful if going forward terms, such as “foster care”, were clearly defined to distinguish between foster care with strangers, foster care with biological family members (kinship care) or residential care setting. Defining exposure more clearly will allow for a greater understanding of the impact of receipt of differing child welfare services on adult mental health trajectories.

Methodological approaches varied widely. In some studies, the use of convenience samples and small sample sizes may have affected the strength of associations. Results from studies that focused on particular sub-populations may not be transferrable beyond these groups. When interpreting results, it should be noted that cohorts were heterogeneous across studies and there was considerable variation in the degree to which studies controlled for potential confounders. Adverse Childhood Experiences (ACEs), such as maltreatment, parental mental ill-health or substance abuse, are associated with poor mental health outcomes and simultaneously influence the nature of child welfare service involvement and placement type. Children placed in OHC are likely to differ on key characteristics compared to those receiving IHC or no services. It is difficult to disentangle the effects of child welfare services from pre-existing adversity, resulting in a high risk of bias. Few studies controlled for ACEs, yet this is vital to develop understanding of the aetiology of mental-ill health in this population. There is a clear need for detailed, population-wide, longitudinal studies which allow for the temporal exploration of risk factors and child welfare service exposure on adult mental ill-health.

The majority of studies examined mental-ill health related to OHC without delineating outcomes by placement type. While these studies show that adults removed from their families are a high-risk group for mental ill-health, OHC is an aggregate concept which masks often complex individual journeys within the child welfare system. It remains important to know which sub-groups may be at particular risk and for which type of condition. The evidence in this study indicates that residential care is associated with poorer adult mental health than other types of OHC, which aligns with findings of studies exploring residential care and mental health outcomes during childhood [95]. The evidence regarding the long-term adult impact of kinship care is mixed, reflecting current literature on childhood outcomes [96].

Future studies should aim to take account of a range of risk factors to meaningfully disentangle casual pathways. Based on this review, results are inconclusive concerning key factors that may contribute, such as age at entry, placement stability or duration in care and meaningful comparison are challenged by the lack of comparable constructs of these measures. Experimental designs are practically and ethically impossible and therefore there is a need to approximate experimental designs using longitudinal data sets and innovative methodological or statistical approaches, such as sibling controls, propensity score matching or instrumental variables to estimate causal effect. There is also a clear need for more research around the long-term mental health outcomes associated with IHC.

## Limitations

Scoping reviews are valuable for providing a broad overview of the published knowledge, key challenges and issues and identifying knowledge gaps. It is important to note that a rigorous quality appraisal of included studies is not a usual feature of scoping reviews because of the breath of their focus. It is possible that not all eligible studies were identified, though the terminology tended to be encompassing in its scope. Limiting the results to papers published in the English language excluded some relevant studies.

## Conclusion

Overall, the results of this review suggest that receipt of OHC or IHC in childhood is associated with an increased risk of poor mental health outcomes in adulthood. However, to date, the literature does not provide adequate understanding as to why this is. Risk factors, such as gender, age at presentation, placement stability, placement type or duration in care, are explored but produce conflicting results. Reason for being known to child welfare services and experience of abuse or neglect is not widely explored or recorded but is necessary to understand underlying causal factors for poor mental health in this population. Care pathways and type of child welfare service received are not well explored. The number of children who receive services at home outweighs children in OHC yet the long-term implications for mental health in this group remains largely unexamined. Future research now needs to focus on the role of specific welfare services, care experiences, placement type and reason for becoming known to child welfare services. There is a need for large-scale, longitudinal studies, for more standardised measures of mental ill-health and more detail from authors on their definitions of “foster care” or “care” exposure.

## Supplementary Information

Below is the link to the electronic supplementary material.

Supplementary file1 (DOCX 22 kb)

Supplementary file2 (DOCX 23 kb)

Supplementary file3 (DOCX 28 kb)

Supplementary file4 (DOCX 25 kb)

Supplementary file5 (DOCX 21 kb)

Supplementary file6 (DOCX 20 kb)

## Data Availability

Unpublished study data are available upon request to the corresponding author.
