# Supplementary material for: Experience of child welfare services and long-term adult mental health outcomes: a scoping review
Source: Soc Psychiatry Psychiatr Epidemiol. 2021 Mar 29;56(7):1115–45. doi: 10.1007/s00127-021-02069-x (PMC8225538; doi:10.1007/s00127-021-02069-x)
Supplement: Supplementary file 1 — Supplementary file1 (DOCX 22 kb) [file 127_2021_2069_MOESM1_ESM.docx]

**Supplementary Figure 1.** Mental health outcomes examined by studies in the review
