# Supplementary material for: Experience of child welfare services and long-term adult mental health outcomes: a scoping review
Source: Soc Psychiatry Psychiatr Epidemiol. 2021 Mar 29;56(7):1115–45. doi: 10.1007/s00127-021-02069-x (PMC8225538; doi:10.1007/s00127-021-02069-x)
Supplement: Supplementary file 2 — Supplementary file2 (DOCX 23 kb) [file 127_2021_2069_MOESM2_ESM.docx]

**Supplementary Table TS1.** Search strategy for databases MEDLINE, EMBASE, PsychINFO, IBSS, Social Policy and Practice

|  | Index headings and keywords (MEDLINE, EMBASE, PsychINFO, Social Policy and Practice) | Keywords  (IBSS) |
| --- | --- | --- |
| 1. Exposure | Child Protective Services OR “child welfare service*” OR “child welfare intervention*” OR “out-of-home-care" OR “in-home-care" OR “looked after child*” OR “foster care” OR “kinship foster care” OR "residential care” | “child protection service*” OR “child welfare service*” OR “child welfare intervention*” OR “out-of-home-care" OR “in-home-care" OR “looked after child*” OR “foster care” OR “kinship foster care” OR "residential care” |
| 1. Outcome | “mental health” OR mental disorders OR “psychiatric disorder” OR “common mood disorder” OR “psychotropic medication” OR psychotropic drugs OR self-injurious behaviour OR self-mutilation OR suicide | \| “mental health” OR “mental disorder*” OR “psychiatric disorder*” OR “common mood disorder” OR “psychotropic medication” “self-harm” OR “suicide” OR “suicidal ideation” \| \| --- \| |
| 1. Outcome time frame | “adult” OR “long-term” OR “lifetime” OR “life course” OR “longitudinal” OR “care leaver” OR “care experienced” OR “alumni” OR “outcome” OR “effect” OR “impact” | “adult” OR “long-term” OR “lifetime” OR “life course” OR “longitudinal” OR “care leaver” OR “care experienced” OR “alumni” OR “outcome” OR “effect” OR “impact” |
| 1. 1 AND 2 AND 3 |  |  |
| 1. Limits | 1995-search date  English language | 1995-search date  English language |
