# Supplementary material for: Experience of child welfare services and long-term adult mental health outcomes: a scoping review
Source: Soc Psychiatry Psychiatr Epidemiol. 2021 Mar 29;56(7):1115–45. doi: 10.1007/s00127-021-02069-x (PMC8225538; doi:10.1007/s00127-021-02069-x)
Supplement: Supplementary file 3 — Supplementary file3 (DOCX 28 kb) [file 127_2021_2069_MOESM3_ESM.docx]

**Supplementary Table TS2.** Final model covariates, included studies of OHC and adult mental health

| Study | Covariates | | | | | |
| --- | --- | --- | --- | --- | --- | --- |
|  | **ACEs** | **Demographics** | **Socio-economic** | **Care experiences** | **Other** |  |
| Anctil et al. (2007), USA | Physical abuse (*ns*) Sexual abuse (- overall mental health)  Neglect (*ns*) Emotional abuse (*ns*) | Gender (*ns*) Current age (*ns*) Ethnicity (*ns*) |  | Age at first placement (*ns*) Intensity of placement change (- no. diagnoses)  Felt loved (*ns*) Helpfulness of foster parent (*ns*) Close relationship with an adult (*ns*) Mental health services (- no. diagnoses)  Independent living services (- overall mental health)  Employment training (*ns*) Tutoring services (*ns*) Education services (*ns*) |  |  |
| Björkenstam et al. (2017), Sweden | Parental death (*ns*) Parental criminality (-) Parental substance abuse (- diagnosis) Parental psychiatric morbidity (-) Parental separation (-) Total no. childhood adversities (-) | Birth year (nr) Gender (nr) Parental country of birth (nr) | Parental education (nr) Household public assistance (-) Residential instability (- diagnosis) |  |  |  |
| Brännström et al. (2017), Sweden |  | Gender (nr) | Parental social class childhood (nr) Household poverty childhood (nr) |  |  |  |
| Bruskas and Tessin (2013), USA | ACEs prior to OHC (-) |  |  | ACEs during OHC (*ns*) Number of placements (*ns*) |  |  |
| Buchanan et al. (2000), UK |  |  | Parental manual class (*ns*) Smoker or drinker (- female) Presence of partner (+ female) Employed (+) Manual class as adult (- female) High Rutter score at 16 (-)  Qualifications (+ female) |  | High non-verbal skills as child (+) |  |
| Cheung and Buchanan (1997), UK |  | Gender (- female) | Childhood social disadvantage (- female age 33) Marital status (+ married; - female no partner at 23, - no partner at 33 years) Qualifications (+) Manual class (- age 23 years) Unemployment (-) Looking after the home (- age 23) |  |  |  |
| Dixon (2006), UK |  | Gender (*ns*) Ethnicity (*ns*) |  | Range of trouble during care (-)  Duration in care (*ns)* Placement movement *(ns)* Age at leaving OHC *(ns)* | M/h problem at baseline (-) Experiencing troubles post-care (-) Intense contact with leaving care worker (-) Other support measures (*ns*) Living in suitable housing (+) Disability (*ns*) |  |
| Dregan et al. (2011), UK |  | Gender (nr) Ethnicity (nr) | Mother's marital status (nr) Parental education (nr) No. of siblings at birth (nr) Socio-economic status in childhood (nr) Mother’s age (nr) Residential area at child's birth (nr) |  | Premature birth (nr) Birthweight (nr) Mother's smoking and drinking during pregnancy (nr) Breastfed (nr) |  |
| Garcia et al. (2012), USA |  | Current age (*ns*) Gender (- female) |  | Placement instability (- Caucasian) Circumstances of exit (*ns*)  Access to independent living services (+ African American) Drug and alcohol services (- Caucasian) Mental health services (*ns*) Tutoring *(ns)* Employment services (- African American) Tangible items for leaving (*ns*) Agency helpfulness (- Caucasian) Subjective preparedness for leaving care (+ Caucasian)  Satisfaction with foster care (+ African American) |  |  |
| Garcia et al. (2015), USA | Parental criminal history (*ns*) Parental mental health (-) Parental substance abuse (*ns*) Chronicity of abuse (-) | Gender (- female) Age (*ns*) |  | Placement instability (-) Agency helpfulness (+ African American/Caucasian) | Childhood mental/physical disability (*ns*) |  |
| Harris et al. (2010), USA |  | Current age (nr) Gender (nr) Ethnicity (nr) |  | Decade entered care (nr) Region served in care (nr) |  |  |
| Patterson et al. (2015), Canada |  | Current age (nr) Gender (nr) Ethnicity (nr) | Level of need (nr) Marital status (nr) |  |  |  |
| Roller White et al. (2009), USA | Maltreatment (nr) Reason for placement (nr) | Age (nr) Gender (nr) Ethnicity (nr) |  | Casey or state OHC (nr) | Childhood mental/physical health problem (nr) |  |
| Roos et al. (2014), Canada |  | Current age (nr) Gender (nr) Ethnicity (nr) | Relationship status (nr) Education (nr) Length of homelessness (nr) |  |  |  |
| Schneider et al. (2009), USA |  | Current age (nr) Ethnicity (nr) |  |  |  |  |
| Teyhan et al. (2018), UK |  | Current age (nr) | Relationship status (nr) Education (nr) Financial difficulties (nr) Social class (nr) Housing tenure (nr) |  | Parity for women (nr) Pregnancy intentional (nr) Pregnancy status/intentions (nr) |  |
| Villegas and Pecora (2012), USA | Maternal m/h problems (-) | Ethnicity (*ns*) Current age (- younger age) Gender (- female) |  | Age at entry (+ younger age) No. of placements (- higher) Maltreatment in OHC (-) Preparedness for leaving (+ high preparedness) |  |  |
| Viner and Taylor (2005), UK |  |  | Paternal social class (nr) Maternal education (nr) Adult social class (nr) |  |  |  |
| Vinnerljung and Hjern (2014), Sweden | Parental substance abuse (nr) Parental m/h problems (nr) | Birth year (nr) Maternal birth country (nr) | Domicile (nr) Maternal education (nr) |  |  |  |
| Wall-Wieler et al. (2018), Sweden |  | Age (nr) | Education (nr) Residency (nr) Employment status (nr) Social welfare receipt (nr) |  | Alcohol misuse (nr) Drug misuse (nr) Psychiatric disorder (nr) Committed a violent crime (nr) Suicide attempt (nr) |  |
| Zlotnick et al. (2012), USA |  | *Total sample*  Gender (- female) Ethnicity (*ns)* | *Total sample*  < federal poverty level (-) < high school education (*ns*) Married (+) Has health insurance (+) |  |  |  |

Notes: nr=not reported; *ns*=not significant; (-) worse mental health outcome; (+) improved mental health outcome
