# Supplementary material for: Experience of child welfare services and long-term adult mental health outcomes: a scoping review
Source: Soc Psychiatry Psychiatr Epidemiol. 2021 Mar 29;56(7):1115–45. doi: 10.1007/s00127-021-02069-x (PMC8225538; doi:10.1007/s00127-021-02069-x)
Supplement: Supplementary file 4 — Supplementary file4 (DOCX 25 kb) [file 127_2021_2069_MOESM4_ESM.docx]

**Supplementary Table TS3.** Final model covariates, included studies of OHC placement type and adult mental health

| Study | Covariates | | | | |
| --- | --- | --- | --- | --- | --- |
|  | **ACEs** | **Demographics** | **Socio-economic** | **Care experiences** | **Other** |
| Benedict et al. (1996), USA |  | Current age (*ns*) Gender (*ns*) Ethnicity (*ns*) |  | Adoption vs other outcome (-) Maltreatment in OHC (-) Maternal drug use and kinship care (-) Mental health problems in OHC (-) | Behavioural problems prior to OHC (-) |
| Carpenter and Clyman (2004), USA | Unwanted 1st sexual experience <18 years (-) | Current age (*ns*)  Ethnicity (+ African American, Hispanic *ns*) | Education (*ns*)  Unemployed (*ns*)  Homemaker (*ns*)  Household income (*ns*)  Poor health status (-) Living alone (-) |  |  |
| Cook-Fong (2000), USA |  | Gender (- female) Ethnicity (*ns*) Current age (+ older age) | Parental education (*ns*) Father's occupational status (-) Education (*ns*) Income (+) Married (+) |  |  |
| Dregan and Gulliford (2012), UK | Placed due to parental illness (-) Placed for abuse/neglect/other/unknown (*ns*) | Current age (nr) Gender (nr) Ethnicity (nr) | Mother's age (nr) Parental social class (nr) Number of siblings (nr) Parental education (nr) | Age at admission (*ns*) Duration in care (- longer stay) Number of placements (-) | Premature birth (nr) Birthweight (nr) Maternal smoking/drinking during pregnancy (nr) Breastfed (nr) |
| Fechter-Leggett and O'Brien (2010), USA | Parent m/health problem (mixed)  Mother physical health problem (+ depression) Placement reason (+ depression)  Type of abuse (- PTSD, - overall m/h) | Gender (mixed) |  | Maltreatment during OHC (mixed) Duration OHC (- any diagnosis) Leaving care resources (mixed) Preparation for leaving care (mixed) Casey region (+ PTSD) School changes (+ panic syndrome) Foster parent helpfulness (mixed) Help with ethnic issues (+ depression) Education services (+ PTSD, anxiety) | Behaviour problems (mixed) |
| Hjern et al. (2018), Sweden |  | Year of birth (nr) Gender (nr) | Area of residence (nr) School marks grade 9 (nr) Income (nr) Labour market participation (nr) |  |  |
| Jackson Foster et al (2011), USA | Sexual abuse (-)  Emotional abuse (-)  Physical abuse *(ns*)  Neglect (*ns*) | Current age (+ younger age)  Gender (- female)  Ethnicity (*ns*) | Poverty *(ns*) | Kinship care x female (+)  Maltreatment during OHC (-)  Placement change rate (*ns*) | Clinical problems (*ns*) |
| Jackson Foster et al. (2015), USA |  | Gender (- female) | Poverty status (+ at or above poverty line) Married (+) | Maltreatment during OHC (-) Felt loved by foster parent (*ns*) Foster parent helpfulness (- less helpful) Close relationship with adult during OHC (*ns*) | Prior behavioural health condition (-) |
| Vinnerljung and Sallnäs (2008), Sweden | History of maltreatment (-) | Gender (*ns*) Immigrant background (*ns*) |  | Age at placement (*ns*) Placed for behaviour reasons (*ns*) Length of placement (*ns*) Placement breakdown (-) |  |

Notes: nr=not reported; *ns*=not significant; (-) worse mental health outcome; (+) improved mental health outcome
