# Supplementary material for: Experience of child welfare services and long-term adult mental health outcomes: a scoping review
Source: Soc Psychiatry Psychiatr Epidemiol. 2021 Mar 29;56(7):1115–45. doi: 10.1007/s00127-021-02069-x (PMC8225538; doi:10.1007/s00127-021-02069-x)
Supplement: Supplementary file 5 — Supplementary file5 (DOCX 21 kb) [file 127_2021_2069_MOESM5_ESM.docx]

**Supplementary Table TS4.** Final model covariates, included studies of OHC and IHC and adult mental health

| Study | Covariates | | | | |
| --- | --- | --- | --- | --- | --- |
|  | **ACEs** | **Demographics** | **Socio-economic** | **Care experiences** | **Other** |
| Berlin et al. (2011), Sweden | Parental substance abuse (nr) Parental psychiatric care (nr) | Gender (nr) Year of birth (nr) | School performance (nr) Maternal education (nr) |  |  |
| Vinnerljung and Ribe (2001), Sweden |  | Current age (nr) Gender (nr) |  |  | Year of risk exposure (nr) |
| Vinnerljung et al. (2006), Sweden | Parental psychiatric illness (nr) Parental alcohol/drug abuse (nr) | Year of birth (nr) Gender (nr) Birth parents' ethnicity (nr) | Maternal socio-economic indicators (nr) Birth mother's residency (nr) |  |  |

Notes: ACE=Adverse Childhood Experience; nr=not reported
