# Supplementary material for: Experience of child welfare services and long-term adult mental health outcomes: a scoping review
Source: Soc Psychiatry Psychiatr Epidemiol. 2021 Mar 29;56(7):1115–45. doi: 10.1007/s00127-021-02069-x (PMC8225538; doi:10.1007/s00127-021-02069-x)
Supplement: Supplementary file 6 — Supplementary file6 (DOCX 20 kb) [file 127_2021_2069_MOESM6_ESM.docx]

**Supplementary Table TS5.** Final model covariates, included studies of IHC and adult mental health

| Study | Covariates | | | | |
| --- | --- | --- | --- | --- | --- |
|  | **ACEs** | **Demographics** | **Socio-economic** | **Care experiences** | **Other** |
| Afifi et al. (2018), Canada | No. of types of child abuse (nr) Frequency of all abuse items (nr) | Gender (nr) Current age (nr) | Marital status (nr) Income (nr) Education (nr) |  |  |
| Vinnerljung et al. (2006), Sweden | Abuse/neglect (-) | Gender (- female) Immigrant background (-) | Teenage mother when 1st child born (*ns*) |  | Anti-social behaviour problems (-) School/psychological problems (*ns*) |

Notes: ACE=Adverse Childhood Experience; nr=not reported; *ns*=not significant; (-) worse mental health outcome; (+) improved mental health outcome
